# Adherence to anti-osteoporosis medication associated with lower mortality following hip fracture in older adults: a nationwide propensity score-matched cohort study

**DOI:** 10.1186/s12877-019-1278-9

**Published:** 2019-10-28

**Authors:** Shan-Fu Yu, Jur-Shan Cheng, Ying-Chou Chen, Jia-Feng Chen, Chung-Yuan Hsu, Han-Ming Lai, Chi-Hua Ko, Wen-Chan Chiu, Yu-Jih Su, Tien-Tsai Cheng

**Affiliations:** 1 Division of Rheumatology, Allergy and Immunology, Department of Internal Medicine, Kaohsiung Chang Gung Memorial Hospital, Chang Gung University College of Medicine, No. 123, Ta-Pei Road, Niao-Sung, Kaohsiung, 833 Taiwan; 2 Chang Gung University College of Medicine, Taoyuan, Taiwan; 3 Clinical Informatics and Medical Statistics Research Center, Chang Gung University College of Medicine, Taoyuan, Taiwan

**Keywords:** Hip fracture, Older adults, Mortality, Osteoporosis treatment, Medication adherence

## Abstract

**Background:**

We investigated the association of anti-osteoporosis medication with mortality risk in older adults with hip fractures and evaluated the influence of medication adherence on mortality.

**Methods:**

We conducted a population-based cohort study and identified a total of 13,123 patients aged 65 years or older with hip fracture from the Taiwan National Health Insurance Database during the period 2001–2010. Individuals with (*n* = 2092) and without (*n* = 2092) receiving anti-osteoporosis medication were matched using propensity score matching (1:1 ratio). The 1-, 3- and 5-year survival rates after the index fracture were compared between patients with and without treatment. In the treated group, survival rate was compared between those with good and non-adherence. Good adherence was defined as the medication possession ratio of ≥80% and non-adherence as a ratio < 80%.

**Results:**

The 1-, 3- and 5-year mortality rates were significantly lower in the treated vs. the non-treated group (all *p* < 0.0001). In the treated group, the estimated 1-, 3- and 5-year survival rates were higher in those with good adherence than in those with non-adherence (all *p* < 0.0001). Regarding all-cause mortality, the adjusted hazard ratio in the treated vs. the non-treated group was 0.63 (95% confidence interval 0.58–0.68, *p* < 0.0001). The good adherence subgroup showed a significantly lower mortality risk than that in the non-adherence subgroup (hazard ratio 0.41, 95% confidence interval 0.32–0.51, *p* < 0.0001).

**Conclusions:**

The 1-, 3- and 5-year survival rates were significantly higher in patients receiving anti-osteoporosis medication than in the untreated group. All-cause mortality rates were lower in patients with good adherence to anti-osteoporosis medication.

## Background

The older population in Taiwan (defined as those aged ≥65 years) is expected to increase from 7.1% of the total population in 1993 to > 14% in 2018 [1]. With an aging population, the occurrence of hip fractures will predictably increase significantly. By 2035, there will be a 2.7-fold increase in the number of hip fractures in Taiwan [2]. Hip fracture rate is highest in Taiwan compared with other Asian countries [3]. Hip fractures have been associated with increased mortality and morbidity in older adults and constitute a high economic burden on patients, families, and the medical community [4, 5]. Thus, Taiwanese health policy makers consider the prevention and treatment of hip fractures in the older people an important issue.

Reportedly, the 1-month, 3-month, 6-month, 1-year, 2-year, 5-year, and 10-year follow-up mortality rates of older Taiwanese patients after hip fracture were 2.5, 6.5, 10.4, 16.3, 25.8, 33.4%, 44.1, and 53.5%, respectively [6]. This high mortality rate has been reported to decline over time; however, not to age- and sex-comparable rates in the general population even 10 years after the fracture [7, 8]. Despite clear evidence demonstrating a considerable effect on health, most patients with osteoporotic fractures remain untreated [9, 10].

Recently, several observational studies and randomized controlled trials have reported that treatment for osteoporosis, particularly with bisphosphonates may improve survival after osteoporotic fractures [11]. However, most studies in a real-world setting have reported that adherence to anti-osteoporosis medication (AOM) is usually lower in clinical practice than that observed in clinical trials [12, 13]. Good adherence to drug therapy is associated with positive health outcomes including a lower risk of fractures [14], lesser utilization of physician and hospital outpatient services, and shorter length of hospitalization [15]. However, the effect of adherence to AOM after a hip fracture on mortality and survival remains unclear. Therefore, the primary aim of this study was to investigate the association between AOM use and mortality in older Taiwanese patients presenting with a hip fracture. The secondary aim of this study was to evaluate the effect of adherence to AOM on short- and long-term post-hip fracture mortality.

## Methods

### Data collection

We used a representative sample of 2 million individuals randomly selected from the entire Taiwanese population in 2000 from the National Health Insurance Research Database (NHIRD) of Taiwan. The NHIRD is managed by the National Health Research Institutes and is released for research purposes. Taiwan launched a single-payer NHI program on March 1, 1995, and by 2007 nearly 99% of the population was enrolled in this program. It is one of the largest nationwide population-based databases in the world. Random samples selected from this database have been confirmed by the NHIRD to be representative of the Taiwanese population. The NHIRD (http://nhird.nhri.org.tw/) contains demographic data of the enrollees, information regarding healthcare professionals and facilities, details of inpatient orders, ambulatory care and expenditure categorized by visits, details of prescriptions dispensed at contracted pharmacies, and a registry of beneficiaries.

### Study design and population

**Fig. 1:**
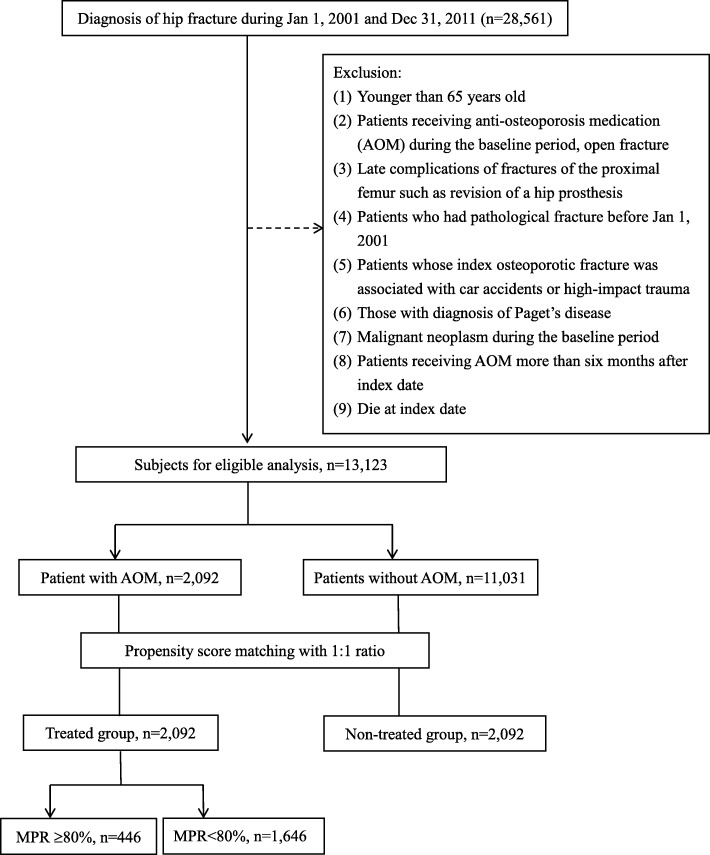
Flowchart showing selection criteria used in enrolling participants

This was a retrospective cohort study, and we enrolled treatment-naive patients aged ≥65 years diagnosed with incident fragility hip fractures based on the International Classification of Diseases, Ninth Revision, Clinical Modification (ICD-9-CM) codes 820.0, 820.00, 820.01, 820.02, 820.03, 820.09, 820.2, 820.20, 820.21, 820.8, 79.15, 79.25, 79.35, or 81.52 between 2001 and 2010. The index date was defined as the date when the patients sustained the hip fracture, and the baseline period was defined as 1 year preceding the index date. Patients were divided into 2 cohorts: patients treated with and without AOM after the index date. The groups were matched using propensity score matching (1:1 ratio). The score was estimated using logistic regression analysis considering age, sex, location, urbanization level, the Charlson comorbidity index (CCI), comorbidities, type of treatment received, and prescribed by medical specialty as variables. We only selected patients with at least 1 osteoporosis-related claim during the baseline period to ensure that the index fracture was related to osteoporosis. Patients excluded from the study were those with any prior osteoporotic hip fracture during the baseline period, patients with conditions that could interfere with the assessment of osteoporotic fractures including those who received AOM during the baseline period, those with open fractures (ICD-9-CM codes 820.1, 820.10, 820.11, 820.12, 829.13, 820.19, 820.9, 820.22, 820.3, 820.30, 820.31, and 820.32), those with late complications of fractures of the proximal femur such as patients who required revision of a hip prosthesis (ICD-9-CM procedure code 81.53), those with pathological fractures (ICD-9-CM codes 733.14 and 733.15) in the preceding year (2000), those whose index osteoporotic fracture was associated with a vehicular accident or high-impact trauma (ICD-9 codes E810-E819, E881-E883, and E8841), those with a diagnosis of Paget’s disease (ICD-9-CM code 731.0), or malignant neoplasms (ICD-9-CM codes 140–208) during the baseline period, and those receiving AOM for > 6 months after the index date. The flowchart showing the criteria for inclusion of patients in this study is shown in Fig. 1. This study was approved by the Ethics Committee of Chang Gung Memorial Hospital (IRB No: 103-2508B), which waived the requirement of informed consent for this population-based cohort study.

### Assessment of adherence

Adherence was calculated based on the medication possession ratio (MPR), which is defined as the ratio of doses available to a patient over a fixed period of time. Good medication adherence was classified as an MPR ≥80% and non-adherence as an MPR < 80% during the first year of AOM treatment.

### Assessment of mortality

The observation period for each enrollee was 5 years after the index fracture or until December 31, 2015, whichever occurred first. All-cause mortality and the date of death were identified from the provincial health care insurance registries and the National Vital Statistics System. Survival time from the index date was calculated. Patients were censored if they were alive at the end of the observation period.

### Assessment of other covariates

We studied other covariates based on the prior literature and/or the clinical rationale for their inclusion. Demographic covariates included age, sex, area of residence, and urbanization level. Clinical covariates included a history of hypertension, diabetes mellitus, heart disease, cerebrovascular disease, chronic kidney disease, chronic liver disease, hyperlipidemia, and chronic obstructive pulmonary disease (COPD). Treatment covariates included the type of hip fracture, type of operation, and AOM prescribed.

### Statistical analysis

Data pertaining to the Taiwanese population provided by the Ministry of the Interior, Department of Statistics (http://www.moi.gov.tw/stat/english/index.asp) were used to calculate the overall mean incidence and the incidence rates of hip fractures between 2001 and 2010, stratified by sex and age. Microsoft SQL Server 2005 was used for data management and computing. All statistical analyses were performed using the SAS statistical software (version 9.3 for Windows, SAS Institute, Inc., Cary, NC). The distributions of categorical sociodemographic characteristics and comorbidities were compared between treated group and non-treated group, and the differences were examined using the *χ*^2^ test for categorical variables and the *t-*test for continuous variables. Cox proportional hazards regression analysis was used to assess mortality rates associated with the use of AOM. Kaplan–Meier analysis was used to estimate survival, and intercohort significance was determined using the log-rank test. Survival time was calculated from the date of the hip fracture to the date of mortality. Multiple regression analysis was performed using a Cox proportional hazards regression model and was reported using 95% confidence intervals (CIs). A two-sided probability value of 0.05 was used to indicate statistical significance.

## Results

Between 2001 and 2010, we identified a total of 13,123 persons aged ≥65 years with hip fractures, including 7929 women (60.4%) and 5194 men (39.6%). We observed that 2029 patients (15.94%) who received AOM and 11,031 patients (84.06%) who did not receive AOM were eligible for analysis (Table 1). After propensity score matching, the present study consisted of 2029 treated and 2029 matched non-treated patients (Fig. 1). Table 1 lists patient characteristics after propensity score matching. All baseline characteristics were well balanced between the cohorts. No statistically significant intercohort differences were observed in age, sex, location, urbanization levels, baseline comorbidities, CCI, and type of treatment received, and prescribed by medical specialty. Treated patients were more likely to have presented with chronic liver disease, and trochanteric hip fractures and to have undergone closed reduction with internal fixation (Table 1). In the treated group, 60% of the patients (1225/2029) received treatment within 1 month after the fracture. The most commonly prescribed AOM was alendronate (*n* = 1202, 57.5%), followed by raloxifene (*n* = 415, 19.8%), and calcitonin (*n* = 372, 17.8%).

**Table 1:** Propensity score-matched baseline characteristics of total study population between treated and non-treated patients

Variables	Total	Treated (*n* = 2092)	Non-treated(*n* = 2092)	*p*-value
Age, mean (S.D.), years	79.1 (7.1)	79.2 (7.0)	79.0 (7.2)	0.3331
Gender, n (%)				0.6212
Male	875 (20.9)	444 (21.2)	431 (20.6)	
Female	3309 (79.1)	1648 (78.8)	1661 (79.4)	
Location, n (%)				0.8364
North	1326 (31.7)	655 (31.3)	671 (32.1)	
Middle	1314 (31.4)	661 (31.6)	653 (31.2)	
Southern	1380 (33.0)	689 (32.9)	691 (33.0)	
East	164 (3.9)	87 (4.2)	77 (3.7)	
Urbanization level, n (%)				0.8062
Metropolitan areas	1296 (31.0)	639 (30.5)	657 (31.4)	
Satellite cities or towns	884 (21.1)	448 (21.4)	436 (20.8)	
Rural areas	2004 (47.9)	1005 (48.1)	999 (47.6)	
Charlson comorbidity index, n (%)				0.0716
0	899 (21.5)	419 (20.0)	480 (22.9)	
1	1034 (24.7)	526 (25.1)	508 (24.3)	
≥ 2	2251 (53.8)	1147 (54.8)	1104 (52.8)	
Comorbidity, n (%)				
Diabetes	1319 (31.5)	672 (32.1)	647 (30.9)	0.4055
Hypertension	2486 (59.4)	1249 (59.7)	1237 (59.1)	0.7056
Cerebrovascular disease	1111 (26.6)	565 (27.0)	546 (26.1)	0.506
Heart disease	1042 (24.9)	531 (25.4)	511 (24.4)	0.4746
Cancer	86 (2.1)	45 (2.2)	41 (2.0)	0.663
Chronic liver disease	261 (6.2)	147 (7.0)	114 (5.5)	0.0349
Chronic obstructive pulmonary disease	792 (18.9)	398 (19.0)	394 (18.8)	0.8746
Chronic kidney disease	370 (8.8)	188 (9.0)	182 (8.7)	0.7439
Hyperlipidemia	581 (13.9)	301 (14.4)	280 (13.4)	0.3478
Type of hip fracture, n (%)				0.0005
Neck (ICD9: 820.00, 820.01, 820.03, 820.8)	1964 (50.2)	975 (49.0)	989 (51.6)	
Cervical (ICD9: 820.0, 820.02, 820.09)	417 (10.7)	186 (9.3)	231 (12.0)	
Trochanteric (ICD9: 820.2, 820.20, 820.21)	1529 (39.1)	831 (41.7)	698 (36.4)	
Type of operation, n (%)				0.0078
Open reduction with internal fixation (ICD 9: 79.35	1230 (60.5)	608 (60.0)	622 (61.0)	
Open reduction without internal fixation (ICD9: 79.25)	8 (0.4)	4 (0.4)	4 (0.4)	
Closed reduction with internal fixation (ICD 9: 79.15)	101 (5.0)	67 (6.6)	34 (3.3)	
Partial hip replacement (ICD-9: 81.52)	694 (34.1)	334 (33.0)	360 (35.3)	
Type of treatment, n (%)				0.8286
Not operated	2151 (51.4)	1079 (51.6)	1072 (51.2)	
Operated	2033 (48.6)	1013 (48.4)	1020 (48.8)	
Prescribed by medical specialty, n (%)				0.6085
Orthopedics	2634 (63.0)	1309 (62.6)	1325 (63.3)	
Non-Orthopedics	1550 (37.1)	783 (37.4)	767 (36.7)	

**Fig. 2:**
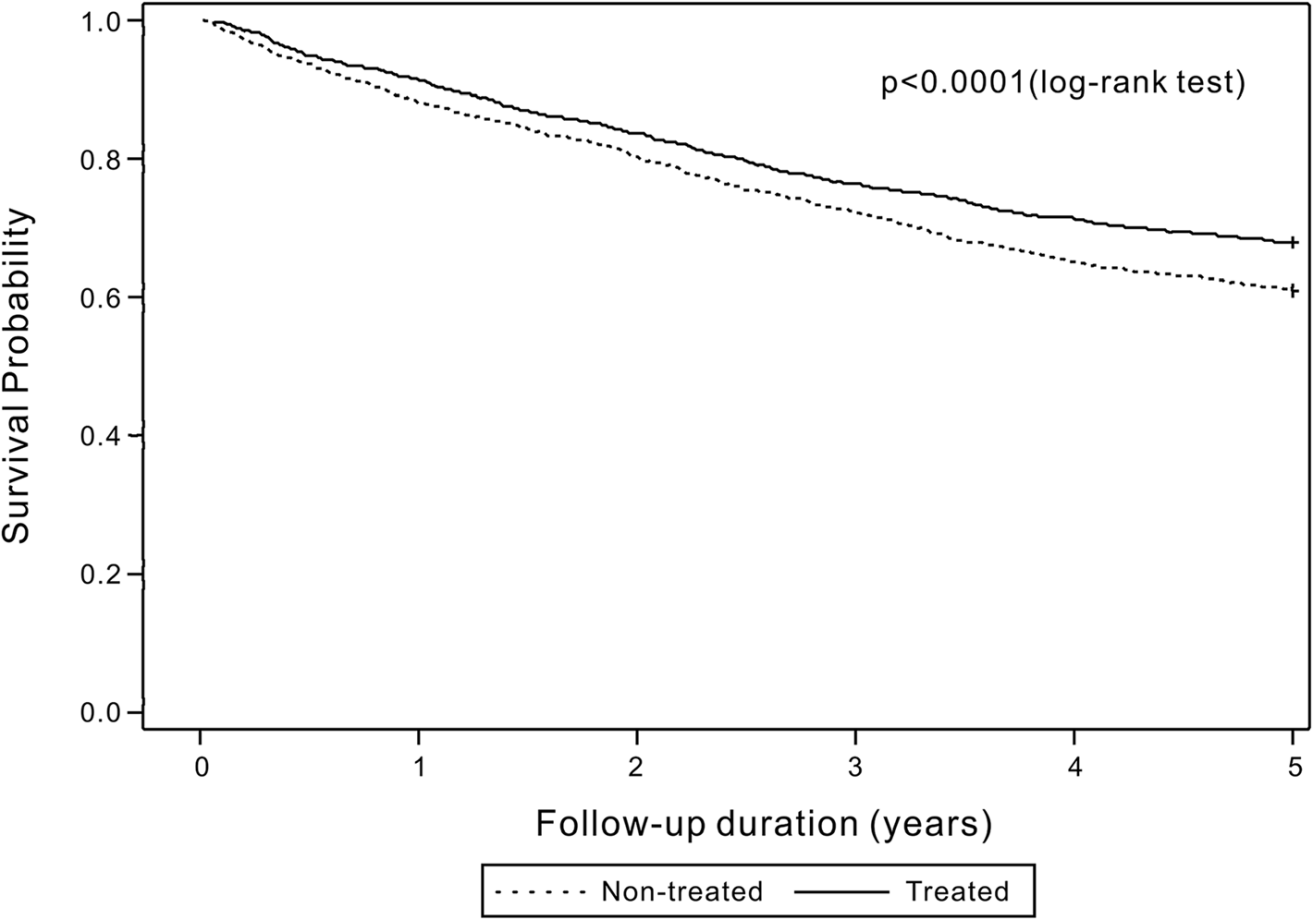
Kaplan–Meier curve of mortality showing a comparison between treated and non-treated patients. Treated patients showed a significantly lower mortality risk than that observed in non-treated patients (log-rank test, *p* < 0.001)

**Fig. 3:**
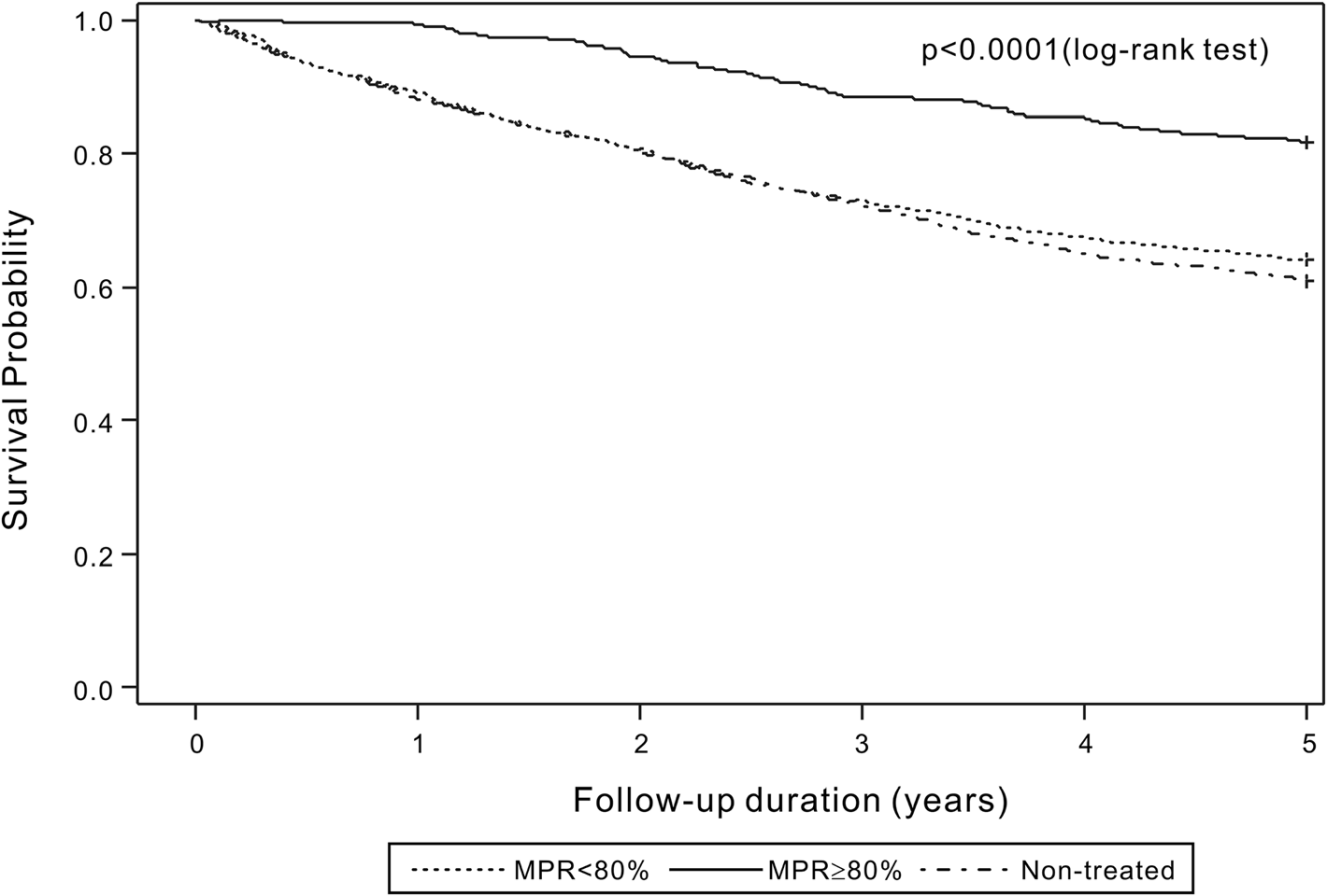
Kaplan–Meier curve of total mortality showing a comparison between patients with good adherence, non-adherence, and non-treated patients. Patients with good adherence showed the lowest risk of all-cause mortality (log-rank test, *p* < 0.001)

The cumulative mortality rates in the treated group at 1-, 3-, and 5 years were 8.6, 23.7, and 32.2%, respectively, compared with 11.8, 27.8, and 39.0%, respectively, in the non-treated group. In the treated group, the cumulative mortality rates at 1, 3, and 5 years were 0.7, 11.4, and 17.7%, respectively, in patients with MPR ≥80% compared with 10.8, 27.0, and 35.7%, respectively, in patients with an MPR < 80%. Kaplan–Meier analysis showed that treated patients demonstrated significantly lower 1-, 3- and 5-year mortality rates than those observed in non-treated patients (log-rank test: all *p* < 0.0001, Fig. 2). The estimated 1-, 3- and 5-year survival rates from the date of the hip fracture were higher in treated patients, particularly in patients with an MPR ≥80% at 1 year (Fig. 3).

The MPR at 1 year was 100% in patients who received zoledronic acid and 60.3, 50.3, 46.7, 41.3, and 14.8% in patients who received ibandronate, teriparatide, alendronate, raloxifene and calcitonin, respectively. The mean MPR of overall AOM use at 1 year was 40.6%. The multivariate-adjusted hazard ratios for all-cause mortality among AOM users (based on an MPR ≥80% or < 80%) compared to patients who never used AOM were 0.41 (95% CI 0.32–0.51), and 0.84 (95% CI 0.75–0.93), respectively (Table 2). Risk factors significantly associated with all-cause mortality were male sex, older age, a high CCI, living in satellite cities or towns and rural areas, and a history of comorbid diabetes mellitus, cancer, COPD, and/or chronic kidney disease (Table 2). In subgroups analysis, we compare the incidence and hazard ratio of mortality stratified by age, gender, comorbidity, and operation according to medication status. Women, aged ≥80 years, patients without comorbidity, or patients who underwent surgery in the treated group showed a significantly lower mortality rate than that in the non-treated group.

**Table 2:** Independent correlates for mortality in elder patients with hip fracture—multivariate time-dependent Cox regression analysis*

	Univariate analysis		Multivariate analysis	
	HR (95% CI)	*p*-value	HR (95% CI)	*p*-value
Osteoporotic drug usage status				
Without osteoporotic drug	Reference group	–	Reference group	–
MPR < 80%	0.91 (0.818–1.011)	0.0804	0.835 (0.748–0.932)	0.0012
MPR ≥ 80%	0.401 (0.32–0.503)	<.0001	0.406 (0.322–0.511)	<.0001
Age	1.062 (1.054–1.07)	<.0001	1.064 (1.055–1.072)	<.0001
Gender				
Male	Reference group		Reference group	
Female	0.746 (0.663–0.839)	<.0001	0.776 (0.684–0.88)	<.0001
Location				
North	Reference group		Reference group	
Middle	1.163 (1.025–1.321)	0.0192	1.066 (0.919–1.236)	0.4006
Southern	0.979 (0.861–1.114)	0.7523	1.067 (0.926–1.23)	0.367
East	1.169 (0.899–1.522)	0.2442	1.047 (0.778–1.408)	0.7639
Urbanization level				
Metropolitan areas	Reference group		Reference group	
Satellite cities or towns	1.146 (0.991–1.326)	0.0657	1.163 (1–1.352)	0.0498
Rural areas	1.202 (1.067–1.355)	0.0026	1.204 (1.05–1.38)	0.0076
Charlson comorbidity index				
0	Reference group		Reference group	
1	1.356 (1.134–1.622)	0.0009	1.266 (1.048–1.529)	0.0146
≥ 2	2.349 (2.018–2.733)	<.0001	1.727 (1.429–2.086)	<.0001
Comorbidity				
Diabetes	1.206 (1.084–1.342)	0.0006	1.242 (1.094–1.409)	0.0008
Hypertension	1.008 (0.908–1.117)	0.8874	0.898 (0.803–1.004)	0.0591
Cerebrovascular disease	1.308 (1.172–1.46)	<.0001	1.063 (0.941–1.2)	0.3256
Heart disease	1.246 (1.113–1.395)	0.0001	1 (0.886–1.128)	0.9996
Cancer	3.621 (2.812–4.662)	<.0001	2.521 (1.907–3.334)	<.0001
Chronic liver disease	1.22 (1.001–1.486)	0.0485	1.194 (0.968–1.471)	0.0978
Chronic obstructive pulmonary disease	1.759 (1.568–1.975)	<.0001	1.311 (1.151–1.493)	<.0001
Chronic kidney disease	1.891 (1.627–2.198)	<.0001	1.787 (1.518–2.104)	<.0001
Hyperlipidemia	0.658 (0.556–0.778)	<.0001	0.629 (0.524–0.755)	<.0001
Type of hip fracture				
Neck	Reference group		Reference group	
Cervical	1.124 (0.944–1.339)	0.1896	1.098 (0.921–1.31)	0.2979
Trochanteric	1.163 (1.041–1.3)	0.0075	1.04 (0.929–1.164)	0.5001
Prescribed by medical specialty				
Orthopedics	Reference group		Reference group	
Non-orthopedics	1.053 (0.948–1.169)	0.3387	0.96 (0.861–1.071)	0.4624

*HR* hazards ratio; *CI* confidence interval

*Stepwise model selection method was used for multivariate analysis

To compare our results with those of other studies as shown in Table 3, ten studies to evaluate the effect of AOM initiated after hip fracture on mortality risk were identified [16–25]. Seven articles (three randomized control trials, two prospective and two retrospective studies) evaluated the mortality of bisphosphonate group and control group. Three articles (two prospective and one nested case-control studies) assess the effect of different AOM classes on mortality. The results showed that AOM (mainly bisphosphonates) can reduce the mortality in older adults with hip fracture. The present study enhances the previous studies’ findings by providing a much more detailed comparison of AOM and post-hip fracture mortality.

**Table 3:** Comparison of anti-osteoporotic medication treatment initiated after hip fracture and mortality risk

Study [Ref. no.]	Country	Design of included studies	No. of patients	Study population	Follow-up durations, means (Y)	AOM choice	Within Time to AOM	Mortality risk (95% CI)
Cree et al. (2003) [16]	Canada	Prospective, Observational	81 (treated) 275 (non-treated)	F and M Age ≥ 65	4Y	HRT, BO, calcitonin, vit-D3	Post-fx 6 M (37%)	0.25 (0.06–1.12) 1-year. 0.34 (0.17–0.70) long-term.
Lyles et al. (2007) [17]	International, multicenter	RCT	1065 (treated) 1062 (placebo)	F and M Age ≥ 50	1.9 Y	Zoledronic acid	Post-op 90D	0.72 (0.56–0.93)
Cecilia et al. (2009) [18]	Spain	RCT	119 (treated) 120 (placebo)	F and M Age ≥ 60	1Y	Alendronate	Post-op 2-4D	0.86 (.29–1.89)
Nurmi et al. (2009) [19]	Finland	Prospective, Observational	103 (treated) 118 (non-treated)	F and M Age ≥ 70 (80.5%)	4Y	HRT, BO, vit-D3, calcitonin, SERM, teriparatide	NA	0.8–0.98
Cameron et al. (2010) [20]	Australia	Nested case-control	83 (treated) 366 (non-treated)	F and M Age ≥ 65	5 Y	BO, HRT, vit-D3, calcitonin	NA	0.20 (0.07–0.55) BO use in the first 3 years Non-BO use: unremarkable
Beaupre et al. (2011) [21]	Canada	Prospective, Observational	101 (treated) 108 (non-treated)	F and M Age ≥ 50	3Y	Alendronate (68%), Risedronate (21%), Etidronate (11%).	Post-fx 1Y (97%), Post-fx 1-2Y (2%), Post-fx 3Y (1%)	0.37 (0.28–0.51) per Year 0.92 (0.88–0.97) per Month
Osaki et al. (2012) [22]	Japan	Prospective Observational	184 (treated) 445 (non-treated)	F Age ≥ 70 (93.6%)	3Y	Risedronate	At the time of discharge from hospital	0.29 (0.04–2.37)
Bondo et al. (2013) [23]	Danish	Retrospective, Observational	1086 (treated) 2147 (non-treated)	F and M Age ≥ 55	3.8Y	BO	Post-fx 4 M	0.76 (0.68–0.85)
Brozek et al. (2016) [24]	Austrian	Retrospective, Observational	2166 (treated) 4332 (non-treated)	F and M Age ≥ 50	4Y	BO	Post-fx 1Y	0.24 (0.15–0.40) 90-days 0.43 (0.36–0.52) 1-year 0.48 (0.42–0.55) 3-year
Cengiz et al. (2016) [25]	Turkey	RCT	56 (treated) 58 (placebo)	F and M Age ≥ 65	1Y	Zolendronic acid	Post-op 2 W	0.41 (0.20–0.86)
Current study (2019)	Taiwan	Retrospective, Observational	2092 (treated) 2092 (non-treated)	F and M Age ≥ 65	5Y	BO, raloxifene, calcitonin, teriparatide	Post-fx 6 M	0.63 (0.58–0.68) Treated 0.42 (0.31–0.52) Adherence 0.84 (0.75–0.93) Non-adherence

*Post-op* post-operative; *Post-fx* post-fracture; *Y* year; *D* day; *M* month; *W* week *BO* Bisphosphonate; *RCT* randomized control trial; *AOM* anti-osteoporotic medication; *CI* confidence interval; *NA* not available

## Discussion

This is the first nationwide cohort study to evaluate the effect of post-hip fracture adherence to osteoporosis treatment on mortality in clinical practice in Taiwan. Our results suggest that compared with no treatment, AOM treatment administered after hip fractures in older patients is associated with lower mortality. However, > 75% of patients showed suboptimal adherence to AOM. Notably, the survival rate of patients with good adherence was higher than that of patients with non-adherence, indicating that poor adherence is associated with an increased risk of mortality.

Reportedly, post-fracture mortality varies significantly based on race/ethnicity, with the risk being lowest in women of Asian ethnicity, followed by women of Hispanic ethnicity [26]. Possible causes for these differences include diverse selection criteria, age and sex distribution, racial differences in bone mineral density, modifiable risk factors, disease management, social relationships, and socioeconomic factors. In the present study, the 1-, 3-, and 5-year follow-up mortality rates were 10.2, 25.7, and 35.6%, respectively. Short- and long-term mortality rates have been reported by several previous studies. In Taiwan, Wang et al. reported similar mortality rates of 16.32, 33.40, and 44.12%, respectively, after osteoporotic hip fractures among inpatients aged ≥60 years [6]. In a study performed in the UK, Haleem et al. reported 6-month and 1-year mortality rates of 11–23% and 22–29%, respectively [27].

Our data indicate that the use of AOM was associated with lower risk of post-hip fracture deaths, which is in agreement with previous studies [11, 16, 17, 19, 21, 23–25]. As shown in Table 3, there are three papers (two studies assessed bisphosphonate, one study considered multiple classes of AOMs) showed the tendency towards lower mortality risk, although it did not reach statistical significance [18, 20, 22]. Most previous observational studies and randomized controlled trials have focused on the association between bisphosphonates and the mortality risk. Data from studies of non-bisphosphonates are fewer. Three previous studies have investigated the associations between multiple drug classes (hormone replacement, bisphosphonates, calcitonin, selective estrogen receptor modulator, and vitamin D3) and mortality [16, 19, 20]. Likewise, we included widely-used osteoporotic drugs (bisphosphonates, calcitonin, raloxifene, and teriparatide) in the treatment group. No study has investigated the effect of denosumab on post-hip fracture mortality. In the literature, there are no studies that have compared the risk of mortality associated with the use of different AOMs after hip fracture. Further investigations are needed to clarify the effect of various AOMs on post-hip fracture mortality reduction.

The mechanism by which deaths are prevented with AOM treatment is not clear and probably multifactorial. Prevention of subsequent fractures is not the only explanation for the observed mortality reduction [28]. Bisphosphonates have anti-inflammatory and immunomodulatory action, and might provide protective effect against cardiovascular events [29–31]. Furthermore, a more plausible interpretation for this association might be that AOM use and mortality are both affected by other factors, and especially by patients’ attitudes and health behaviors. Patients are more likely to die following a hip fracture if they are the sort of people who do not accept medical advice, who do not accept or persist with offered treatment, or have personalities and social status that encourages their doctors to believe that they will not cooperate with AOM, or with other measures which their doctor might consider potentially useful in improving their health after hip fracture. Further research will hopefully clarify our understanding of the reduction in mortality risk observed with AOM use after hip fracture.

Based on the literature, under-treatment of osteoporosis is common in older patients with hip fractures [9]. Initiating treatment is an essential first step in the management of osteoporosis; however, several patients do not continue anti-osteoporosis treatment after the diagnosis. A recent study from the US reported that < 20% of women with a first fragility fracture received treatment for osteoporosis in the first year after the fracture [32]. In this study, we observed that a hip fracture precipitating the diagnosis of osteoporosis was no guarantee of adherence to subsequent AOM treatment, and only 15.9% of the patients received a first prescription of AOM within 6 months after the hip fracture. This is in agreement with previous studies, suggesting low prescription rates for anti-osteoporosis therapy in patients with fractures, as well as low adherence [33–35].

Owing to minimal exercise after a hip fracture, the loss of bone mineral density has been reported to be 2% during the first 2 months and 4–7% during the first year post-fracture [9]. Thus, prevention of bone loss warrants the institution of post-fracture AOM as early as possible [35]. Timely initiation of AOM for older patients with hip fracture is critical for achieving optimal treatment outcomes. As shown in Table 3, the time between hip fracture diagnosis and starting AOM represents substantial heterogeneity between studies. Lyles et al. reported that initiating zoledronate therapy within 90 days after surgical repair was associated with improved survival [17]. Cengiz et al. confirmed that the use of zoledronic acid in the second postoperative week in older patients was a safe treatment modality to reduce mortality and improve functional outcomes [25]. Some studies showed that patients receiving bisphosphonate within 1 year after hip fracture had significant reduction in mortality [21, 24]. In the present study, we demonstrated that initiating AOM therapy within 6 months after the hip fracture is associated with lower mortality. This finding is in agreement with previous studies regardless of the population or the time of initiation of AOM treatment.

Comparing adherence rates across published studies is difficult owing to differences in study methodologies, enrolment criteria, definitions of adherence, mono/sequential therapy, and the length of the study [36]. Lin et al. performed a pharmacoepidemiological study using the NHIRD in Taiwan and reported that only 38% of patients > 50 years of age continued to show good compliance during the first year of alendronate therapy [37]. However, in the present study, we observed that the adherence rate in older adults was only 21.3%. This finding could be attributed to poor adherence rates among older patients [38], which in turn could be related to patients’ health beliefs, polypharmacy, complicated drug regimens, decline in memory, and concerns over adverse effects, or a poor understanding of their illness, and the consequences of under-treatment [39].

Studies assessing the link between medication adherence and clinical outcomes have consistently demonstrated that better adherence is associated with positive outcomes and may be a surrogate marker for overall health behavior [40, 41]. Simpson et al. reported that adherence to drug therapy was associated with reduced mortality [40]. We observed an approximately 10–18% increase in the short- and long-term risk of death among patients with poor adherence to AOM compared to those with good adherence. The lower mortality risk in the good adherence group could be attributed to: 1) Reportedly, the re-fracture rate among inpatients with an MPR ≥80% is significantly lower than that in patients with an MPR < 80% [42], and a reduction in re-fracture rates is shown to be significantly associated with a lower mortality risk, 2) patients included in observational studies are not randomly assigned to the exposure groups; thus, the observed differences in outcomes between adherence groups may be secondary to differences in the patients’ overall adherence behavior including lifestyle and other unmeasured confounders rather than differences in their exposure to medication, 3) empirical support for the effect of good adherence was reported among women participating in clinical trials involving hormone and bisphosphonate therapy, in which adherence to a blinded placebo was shown to confer a significant benefit on mortality [23] and, 4) causes of death after hip fractures are predominantly related to infections including pneumonia and septicemia [43]. Poor adherence to AOM was significantly associated with an increased infection rate [44]. Patients treated with zoledronic acid following a hip fracture were less likely to die of pneumonia than patients treated with placebos [45]. Therefore, adherence to AOM would reduce infection-related deaths, which is perhaps attributable to AOM-mediated immune defense against infections [29, 30, 46].

The use of a population-based dataset comprising a large number of subjects could be considered the strength of this study. However, the limitations are: 1) Data regarding a few potentially important confounders such as bone mineral density, bone turnover markers, smoking status and health behavior were not available in the NHIRD. Nutritional supplements are not covered by the NHI program in Taiwan; thus, data regarding vitamin D and calcium supplementation were lacking. 2) We defined medication adherence using drug dispensing records. Thus, we are unsure whether the drugs were actually consumed and also whether optimal drug doses had been administered to patients. 3) Owing to the observational design, this study did not consider different modalities of care delivered to patients. 4) Administrative claims data can be incomplete or contain inaccurate coding of diagnoses and comorbidities, and different administrative databases can contain different rates of comorbidities.

Our results highlight the importance of adherence to post-hip fracture secondary prevention and that AOM is associated with marked benefits. Therefore, we emphasize that patients must adhere to AOM to fully benefit from treatment and that an MPR of ≥80% can be used as an effective parameter to define good adherence. However, further investigations are needed to elucidate the reasons associated with a reduction in the mortality risk. Population-based, randomized, placebo-controlled trials with overall mortality as the main endpoint should be performed to verify our results.

## Conclusions

To conclude, the results of the present investigation demonstrate that short- and long-term mortality rates after hip fractures were lower among patients using AOM, particularly among those with an MPR ≥80%.

## Data Availability

The data that support the findings of this study are available from National Health Insurance Administration Ministry of Health and Welfare in Taiwan but restrictions apply to the availability of these data, which were used under license for the current study, and so are not publicly available. Data are however available from the authors upon reasonable request and with permission of National Health Insurance Administration Ministry of Health and Welfare in Taiwan.

## Abbreviations

AOM: Anti-osteoporosis medication
CCI: Charlson comorbidity index
CI: Confidence interval
COPD: Chronic obstructive pulmonary disease
HR: Hazard ratio
ICD-9-CM: International Classification of Diseases, Ninth Revision, Clinical Modification
MPR: Medication possession ratio
NHIRD: National Health Insurance Research Database
